# Botulinum Toxin Effects and Its Association with Vitamin and Mineral Supplementation: A Narrative Review

**DOI:** 10.3390/nu18030491

**Published:** 2026-02-02

**Authors:** Ema Puizina, Dinko Martinovic, Slaven Lasic, Lovre Martinovic, Jasna Puizina, Emil Dediol, Slaven Lupi-Ferandin, Josko Bozic

**Affiliations:** 1 Department of Maxillofacial Surgery, University Hospital of Split, 21000 Split, Croatiadinko.martinovic@kbsplit.hr (D.M.); 2 Department of Maxillofacial Surgery, University of Split School of Medicine, 21000 Split, Croatia; 3 Department of Neurology, University Hospital Dubrava, 10000 Zagreb, Croatia; 4 Medical Studies, University of Split School of Medicine, 21000 Split, Croatia; 5 Faculty of Science, University of Split, 21000 Split, Croatia; 6 Department of Maxillofacial Surgery, University Hospital Dubrava, 10000 Zagreb, Croatia; 7 Department of Pathophysiology, University of Split School of Medicine, 21000 Split, Croatia

**Keywords:** botulinum toxin, aesthetic medicine, oral supplementation, minerals, vitamins

## Abstract

**Background:** With the emerging global popularity of botulinum neurotoxin (BoNT), both for aesthetical and medical purposes, there is a rising need for achieving better outcomes. The aim of this narrative review was to comprehensively cover the effect of BoNT, as well as its possible interactions with everyday mineral and vitamin supplementation. **Results:** It is well established that BoNT exerts its paralytic effects through zinc-dependent cleavage of SNARE proteins, blocking acetylcholine release at the neuromuscular junctions. However, after meticulous research of the available literature regarding the effect of oral supplementation on BoNT, there is very scarce data. The effect of zinc supplementation on the duration and effectiveness of BoNT in some facial applications was studied in a couple of clinical studies; however, systematic reviews indicate inconsistent results. Copper acts as a noncompetitive inhibitor, potentially antagonizing BoNT in animal models. Magnesium and calcium exhibit theoretical synergistic or compensatory roles via neuromuscular transmission modulation but lack clinical validation. Vitamin B complex shows no interference in rat studies and vitamin D influences baseline muscle strength and dosing needs, while vitamin E increases bruising risk but not efficacy. **Conclusions:** Even though zinc supplementation holds promise for potentiating BoNT effects, evidence for both zinc and other supplements remains speculative or contradictory, underscoring the need for randomized controlled trials to develop evidence-based guidelines. Clinicians should assess patient supplementation status pre-treatment to optimize outcomes and minimize complications, particularly advising against high-dose vitamin E peri-procedurally.

## 1. Introduction

Botulinum toxin (BoNT) is widely applied across modern clinical practice, spanning cosmetic procedures as well as treatments for overactive muscle tone, autonomic disturbances, and chronic pain conditions [1].

The BoNT attribute of “a miracle poison” is rightly justified, as both clinical and cosmetic applications are rising with each decade due to novel insights. With the continued expansion of BoNT indications, many patients also consume oral nutritional supplements, frequently on their own initiative to improve aesthetic outcomes, promote skin health, or address concurrent medical issues. Despite this trend, the scientific literature offers limited and often inconsistent guidance on whether such supplements enhance BoNT efficacy, interfere with its therapeutic effects, or create additional safety challenges. Hence, an examination of its role in current medical practice and aesthetic purposes, combined with the enhancement of the effect by micronutrient supplementation, is essential in optimizing treatment outcomes and ensuring patient safety.

A meticulous bibliographic search of electronic databases was conducted up to 1st November 2025. A filter relating to the language of the publications was used and all studies in a language other than English were excluded. The search was performed on the PubMed (MEDLINE), Scopus, Web of Science and Cochrane electronic databases. Moreover, a search of the gray literature and on Google Scholar was also performed, and the bibliographic sources of previous systematic reviews on the subject were correspondingly investigated. The following terms were used to search the databases: “botulinum toxin”, “botox”, “BoNT”, “dietary supplements”; “supplementation”; “mineral”; “vitamin”; “zinc”; “magnesium”; “calcium”; “copper”; “vitamin B”; “vitamin C”; “vitamin D”; “vitamin E”. The terms were combined using the commands “AND” or “OR”. The inclusion criteria were studies that indicated and reported data regarding effects of dietary supplementation with BoNT. The exclusion criteria were studies published in a non-English language and case reports.

### 1.1. Botulinum Toxin Main Characteristics

BoNT is the most potent neurotoxin known to humanity and has a wide spectrum of therapeutic and cosmetic indications. It is produced by *Clostridium* bacteria from the *Clostridiaceae* family [2]. The toxin is generally found in nature, in the form of spores, and is most commonly associated with botulism, a condition that occurs after ingestion of contaminated food, wound infection or colonization of the gastrointestinal tract in infants.

It was Justinus Kerner who first examined the food poisonings due to spoiled ham and sausage products in 1800s that led to the hypothesis of BoNT. Even then, he presumed the usefulness of BoNT-induced flaccid paralysis and autonomic symptoms as a potential treatment for hyperkinetic movement disorders and hypersecretion of bodily fluids. Emile Pierre-Marie van Ermengen isolated the bacterium responsible for BONT production and named it Bacillus botulinum around 1895. From the 1920s to the 1970s, a series of experiments, from the crystallization of its toxin structure to its blocking of the neuromuscular junction transmission, solidified its predictable effect as a therapeutic. This led to the first FDA approval in 1989 for the use of BoNT in treating strabismus, blepharospasm, and hemifacial spasm in patients under 12 years old [3]. From then on, FDA-approved indications for BoNT-A treatment surged for both treatment and cosmetic purposes. The primary effect of transient chemical denervation of muscles was expanded upon the realization of BONT’s effect on blocking the release of other neurotransmitters, channels to include treatment of pain disorders, and exocrine hypersecretion, among others.

There are multiple serotypes of botulinum toxins A-G, varying in molecular size, biosynthesis and cell mechanisms, and type A is established as the most potent one. The human nervous system is susceptible to five toxin serotypes (BoNT-A, B, E, F, G) and unaffected by two (BoNT-C, D) [3]. Several botulinum toxin-A products are available worldwide: abobotulinumA (Dysport^®^, Ipsen Pharmaceuticals), onabotulinumtoxinA (Botox^®^, Allergan Inc.), prabotulinumtoxinA (Neuronox^®^, Evolus, Inc.) and incobotulinumtoxinA (Xeomin^®^, Merz Pharmaceuticals) are most commonly used [4]. Type B, rimabotulinumB (Myobloc/Neurobloc^®^, US-WorldMed-Solstice), is also available for medical purposes [5,6]. The need for improved effect duration, lower immunogenicity and better selectivity led to the creation and testing of novel BoNT formulations such as recombinant BoNT, chimeric BoNT, non-Clostridial BoNT, and BoNT with modified target selectivity [6]. However, the therapeutic indications of BoNT may become the victims of its success, with manufacturers focusing on cost–revenue, leading to the exclusion of potential new clinical studies for even more novel indications [7].

**Figure 1:**
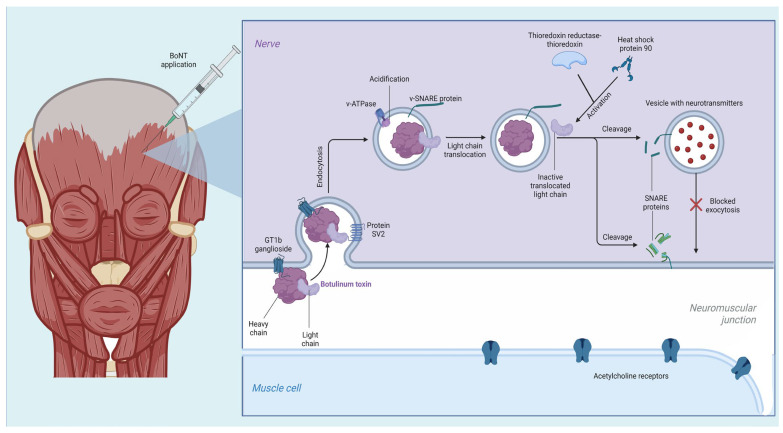
Pathophysiological mechanism of BoNT action.

An understanding of the molecular and cellular mechanisms of botulinum toxin action is fundamental for interpreting the potential effects of nutritional supplements on treatment outcomes [1]. BoNT consists of two peptide chains connected with a disulfide bridge. The heavy chain (100 ~kDa) is responsible for the adherence and translocation of the enzymatic zinc-containing protease light chain (50 ~kDa), which is accountable for the effect of BoNT [8]. After application, the heavy chain connects to the gangliosides of the synaptic neuron membrane such as GT1b [9]. However, BoNT also requires the presence of transmembrane proteins such as synaptic vesicle protein 2 (SV2) and/or synaptotagmin (Syt) to start the endocytosis process and later translocate the light chain into the cell. These proteins are abundantly present in motor neurons, hence the specificity of BoNT for motor neurons. Furthermore, it is important to highlight that BoNT serotypes differ in the specificity to different anchors for the heavy chain [1]. After the endocystosis, vesicular ATPase conducts acidification of the vesicle, which in turn helps to translocate the light chain out of the vesicle. However, during the translocation the light chain is still in its inactive folded form and is then activated with catalytic enzymes such as heat shock protein 90 and thioredoxin reductase-thioredoxin. The active light chain cleaves the SNARE (soluble N-ethylmaleimide-sensitive factor attachment protein receptor) proteins: SNAP25, synaptobrevin, and syntaxin [10]. Since these proteins are essential for the adherence of the intracellular vesicles and neuron membrane, this cleavage leads to the blockade of the exocystosis process (Figure 1) [6].Thus, BoNT presynaptically blocks the release of acetylcholine (ACh) at the neuromuscular junction from the axon terminals of motor neurons, preganglionic sympathetic and parasympathetic neurons and postganglionic parasympathetic nerves that differ slightly for each serotype [11]. ACh is the leading neurotransmitter at the neuromuscular junction, where it activates ACh/nicotinic receptors on the muscle cell membrane. An action potential-induced merger of ACh-containing vesicles and exocytosis is thus blocked, preventing the transmission of the contraction signal to the muscle cell. This leads to transient chemical denervation or muscle relaxation. Moreover, ACh serves as the neurotransmitter in certain postganglionic sympathetic fibers instead of norepinephrine, particularly those regulating sweat gland secretions, which consequently leads to anhydrosis. Additionally, some postganglionic parasympathetic fibers use ACh to control salivary gland secretions. Beyond its direct action on target organs, ACh also modulates neuroactive amino acids, a dual function that has contributed to the broadening of BoNT’s clinical applications [12].

BoNT inhibits the release of larger molecules responsible for pain signaling, such as CGRP, PACAP, substance P, and sensory receptors such as TRPV1. Also, BoNT inhibits the release of catecholamines from chromaffin cells, ATP and glutamate from glial cells, and TNF from monocytes, among others [1,6,7]. This effect is responsible for a myriad of clinically useful effects.

### 1.2. Duration of BoNT Action

BoNT’s effect is temporary, starting around 24–72 h after treatment and lasting up to 3 or 6 months, depending on the serotype, treated indication, dose, injection technique and patient response. The duration of the effect of BoNT is defined by the internalization time of BoNT, the half-life of BoNT, the turnaround of SNARE proteins, and axonal sprouting [13,14]. The temporary effect of BoNT implies repetitive intramuscular toxin application.

Repetition of toxin application may lead to an immunological response by neutralizing antibodies, and with time to treatment failure. BoNT heavy and light chains form a stabilizing complex with neurotoxin-associated proteins (NAPs) such as hemagglutinin proteins, and Non-toxin Non-hemagglutinin Activity (NTNHA) proteins. Both BoNT and NAPs, as proteins, are recognized by the immune system as foreign antigens, with NAPs appearing to stimulate the immune response.

The excipient used to stabilize the toxin, human serum albumin (HSA), might also induce an immune response [15]. OnabotulinumtoxinA (Botox^®^, Allergan Inc.) has the lowest incidence of neutralizing antibodies with the highest incidence of abobotulinumtoxinA (Dysport^®^, Ipsen Pharmaceuticals) and then incobotulinumtoxinA (Xeomin^®^, Merz Pharmaceuticals). Easy-to-use clinical tests for treatment failure, such as the unilateral brow injection test and the frontalis antibody test, can replace formal, expensive and complicated bioassays such as the mouse protection assay (MPA) and mouse hemidiaphragm assay (MHDA). Production process, NAPs, excipient use, BoNT quantity (potency units U), and reconstitution requirements hinder the interchangeability of doses between different BoNT formulations. However, treatment failure due to neutralizing antibodies necessitates the trial of a different formulation. Clinical practice supports these ratios: onabotulinumtoxinA:incobotulinumtoxinA = 1:1, onabotulinumtoxinA:abobotulinumtoxinA = 1:2.5, and onabotulinumtoxinA:rimabotulinumtoxinB = 1:50 [16].

### 1.3. Adverse Effects and Complications

BoNT’s adverse effects and complications are divided into those localized to the injection area, complications due to unwanted spreading of BoNT and systemic complications.

Most local adverse effects are mild, transitory, and self-limited. They typically emerge within days after injection and resolve spontaneously without the need for further intervention. Pain, bruising, hematoma, local site reactions and infections are the most common and they are all symptomatically treated. Hematoma is a complication related to vascular injury and may occur immediately after injection, sometimes persisting for an extended period. Prompt management with local compression and application of pressure and ice can reduce bruising and ecchymosis. Preventive measures include pre-treatment cooling to induce vasoconstriction, screening for coagulation disorders and advising patients to discontinue medications and supplements that impair clotting such as aspirin, NSAIDs, and vitamin E 10–14 days prior to treatment [17].

Faulty technique or excessive BoNT dosage leads to unwanted spreading distal to the injection site, causing ptosis, facial weakness, dysphagia, dysarthria, respiratory infection, other muscle weakness, acute urinary retention and urinary infection. Prescreening of patients, avoidance of excessive dosage uses and correct needle placement with the help of ultrasound or electromyography prevent these complications.

Systemic complications such as anaphylaxis and systemic botulism are exceedingly rare. Common mild adverse reactions to BoNT-A include nausea, fatigue, malaise, flu-like symptoms and rashes at sites distant from the injection area, while the most frequent clinically significant systemic adverse effect is unintended muscle weakness that may interfere with daily activities [18]. Patients allergic to BoNT should be excluded, alongside those with neuromuscular disorders and those patients using aminoglycosides, anticholinergics, and muscle relaxants.

Studies have demonstrated that frequent administration of BoNT-A at intervals shorter than 12 weeks or the use of cumulative doses exceeding 300 units is associated with an increased risk of neutralizing antibody formation, which can consequently reduce therapeutic efficacy [19]. Treatment failure due to neutralizing antibodies can be prevented using lower BoNT dose and longer intervals between injections [20].

### 1.4. BoNT in Current Medical Practice

Treatment with botulinum toxin is effective for many clinical conditions involving involuntary muscle activity or increased muscle tone, such as dystonia, spasticity, overactive bladder, and migraines [5]. BoNT gained recognition from the cosmetic industry regarding dynamic wrinkle treatment and has expanded in aesthetic purposes such as muscle size reduction, impact on skin quality or excess sweating. Besides aesthetic uses, therapeutic uses of BoNT are most significant in the field of neurology. The FDA approved onabotulinumtoxinA (Botox^®^, Allergan Inc.) treatment for cervical dystonia in December 2000 and for spasticity due to traumatic brain injury or stroke in March of 2010; in October 2010, the FDA approved its use in treating chronic migraine. Other formulations of BONT soon followed [1].

FDA-approved BoNT indications in neurology are for cervical dystonia, hemifacial spasm, blepharospasm, spasticity, and chronic migraine. Off-label use of BoNT in neurology includes for tremor, tics, other forms of focal dystonia, palatal myoclonus, myokymia, bruxism, tension headaches, trigeminal neuralgia, myofascial pain, and brachial plexus injury [13,20]. BoNT is consistently effective in movement disorders, with improvements ranging from 60 to 90% and effects usually starting in the first two weeks and lasting for three to four months. The response to treatment is usually long-lasting, with local and manageable side effects [21]. Treatment failure is usually 1%, while in cervical dystonia it may be up to 6.5% [13]. Detailed analysis of BoNT’s effect on movement disorders is beyond the scope of this review; therefore, the reader is directed to an expert review by Anandan and Jankovic [21]. Spasticity, independently of diagnosis (multiple sclerosis, stroke, traumatic brain injury, cerebral palsy), has a predictable good response to BoNT. However, for full effect, early intervention and physical rehabilitation are a prerequisite [1]. The interplay between cosmetic and medical uses of BoNT led to the discovery of headache improvement in patients in whom BoNT was used for facial aesthetic purposes. Based on PREEMPT trials, BoNT is now FDA-approved as an effective prophylactic treatment for chronic migraine, while BoNT use in trigeminal neuralgia, trigeminal autonomic cephalalgias, and nummular headaches is still off-label [22]. Use of BoNT in orofacial pain and disorders, besides sialorrhea, although highly effective, is also off-label [23]. Chemical denervation of muscles has expanded over the years to smooth muscles with the FDA approval of BoNT use in adult and pediatric neurogenic detrusor overactivity and non-neurogenic overactive bladder. Myriad potential off-label indications in gastroenterology and urology/gynecology followed. Some of these indications are for treatment of achalasia, gastroparesis, ineffective esophageal motility, feeding disorders, chronic anal and rectal fissures, proctalgia fugax, prostatic obstruction, outflow obstruction syndrome, vaginismus, vulvodynia, chronic pelvic pain, and painful bladder syndrome [13,20]. Ironically, strabismus as an initial indication for BoNT is more often replaced by definite surgical correction due to the transitory effect of BoNT [20].

BoNT treatment is expensive, ranging from USD 10 to 20 per unit of onabotulinumtoxinA (Botox^®^, Allergan Inc.) to USD 4–8 per unit of abobotulinumA (Dysport^®^, Ipsen Pharmaceuticals), especially considering the need for repetitive toxin, e.g., cation. For example, blepharospasm requires 1.25–2.5 U in each of the three sites per eye. Sialorrhea is treated with 100 U across 4 sites and chronic migraine with 155 U to 195 U across 31 to 39 injection sites, while cervical dystonia requires 200 U to 300 U injected in different muscles. Spasticity necessitates the use of the maximum tolerable BONT dose of 400 U across affected muscles (h). This leads to inequality in social healthcare reimbursements, influencing BoNT indications. Beyond its established role in managing medical conditions, BoNT has become a cornerstone of aesthetic medicine.

### 1.5. BoNT for Aesthetic Purposes

Aesthetic usage of BoNT started in 1992 when Alastair Carruthers published a paper reporting that BoNT injection removed glabellar wrinkles [24]. Over the last three decades, BoNT has been recognized as a cornerstone in aesthetic medicine, primarily because it offers a minimally invasive and reversible approach to smoothing facial wrinkles and lines [25,26].

Although it is primarily used in aesthetics for wrinkle therapy, there are some other purposes like hyperhidrosis of axilla, palms and scalp, body shape contouring, facial asymmetry and skin quality improvement. Aesthetically, it is labeled for use on forehead wrinkles, glabellar and periorbital region. Furthermore, common off-label aesthetic uses include jawline slimming, neck and shoulder contouring, nasal reshaping, treatment of gummy smile, scar modulation and the control of both sebaceous and sweat gland activities [26]. Despite growing clinical adoption, many of these indications lack standardized dosing and long-term evidence, highlighting the importance of practitioner expertise and further research.

Modern aesthetic medicine is achieving optimal results with BoNT-A, analyzing the dynamic physiology of face and neck muscles. For example, BoNT-A is used for “gummy smile” or gingival smile due to excessive vertical maxilla height, hyperfunction of the lip elevator and delayed passive eruption; the treatment of levator labii superioris alaeque nasi muscle offers good cosmetic and functional results regarding gingival display [27]. Another useful example of BoNT-A usage is platysma muscle relaxation for improving the mandibular line and decreasing marionette lines, together with the depressor anguli oris muscle [28]. Moreover, BoNT-A is administered into facial expression muscles to treat habitual or hyperactive facial wrinkles [29]. Repeated administration of BoNT-A may lead to atrophy of facial expression muscles; however, due to their thin structure, such changes are often difficult to detect clinically, which is in contrast to the more apparent atrophy observed in thicker muscles such as the masseter or trapezius. Therefore, by leveraging the muscle atrophy that may result as a side effect of BoNT-A treatment, noninvasive correction of pathological muscle hypertrophy and cosmetic contouring can be achieved [30]. These procedures include lower face contouring for masseter hypertrophy through injection into the masseter muscle, as well as neck and shoulder contouring by inducing motor paralysis of the trapezius muscle [31].

A systematic review and meta-analysis reported that intralesional BoNT-A injections were superior to intralesional corticosteroids and a placebo for managing hypertrophic scars and keloids [32]. The therapeutic rationale extends beyond reducing dynamic tension around the scar through localized chemodenervation; it seems plausible that BoNT-A may also act directly on fibroblasts by influencing apoptotic, migratory and profibrotic signaling pathways. By weakening nearby musculature, BoNT-A can lessen mechanical stress during wound healing and it may additionally dampen the inflammatory processes associated with increased cellular metabolic activity that contribute to scar overgrowth. Research has shown that BoNT-A influences fibroblast behavior by downregulating transforming growth factor-β1 and connective tissue growth factor, thereby limiting fibroblast proliferation [33]. Furthermore, it modulates the fibroblast cell cycle and inhibits the transition of fibroblasts into myofibroblasts [33]. Despite these findings, further high-quality studies are required to clarify the specific role of BoNT-A in treatment algorithms for hypertrophic and keloid scars.

Effects on skin quality consist of improvements in sebum production, pore size, erythema index, skin texture and elasticity [34]. It is worth noting that diluted BoNT-A can be injected into the dermis in the form of mesobotox or microbotox. Effects on skin quality consist of improvements in sebum production, pore size, erythema index, skin texture and elasticity [35]. However, the scientific basis for mesobotox in facial complexion improvement remains limited. Its proposed effects are attributed to mild muscle paralysis, resulting in subtle lifting via depressor muscle inhibition, wrinkle reduction, and improved skin texture through erector pili paralysis and minor lymphatic drainage impairment [36].

## 2. Oral Supplementation Correlation with BoNT

Due to the aforementioned high popularity of BoNT, both for aesthetical and medical purposes, there is a high necessity to further refine its effects to achieve better outcomes. Since dietary supplements have also become very popular among the general population, it is necessary to research the possible interplay between them and BoNT. Even though there is still a lack of evidence regarding the correlation between BoNT and the most common dietary supplement, this section provides both established and mechanistically hypothesized interactions (Table 1).

### 2.1. Zinc Supplementation

Zinc is an essential trace element and a critical micronutrient for human metabolism, functioning as both a structural and catalytic component in numerous enzymes. It participates in diverse physiological processes including cell growth and division, gene expression, DNA metabolism and repair, protein synthesis and immune system regulation [57]. Zinc homeostasis is tightly regulated at multiple biological levels through zinc transporter families. The body maintains approximately 2–4 g of zinc distributed throughout tissues, with the highest concentrations in the brain, muscle, skeletal system, liver, kidney and reproductive organs. Given the body’s inability to store zinc in significant quantities, regular dietary intake is essential to maintain adequate levels and prevent deficiency [58]. Beyond its fundamental role as an enzymatic cofactor, zinc exerts critical effects on immune cell development and function, bone homeostasis through regulation of osteoblast and osteoclast activity, central nervous system development and synaptic plasticity, antioxidant defense mechanisms and inflammatory signaling pathways.

As aforementioned, BoNT-A effect develops through cleavage with zinc-dependent proteolysis of the SNARE protein which is responsible for binding vesicles with AcH to cellular membrane and consequent release of the neurotransmitter [59]. Moreover, the biological activity of BoNT-A is dependent on body zinc reserves, as zinc is essential for toxin activation and for the proteolytic cleavage of SNARE [37].

A double-blind, placebo-controlled pilot study concluded that supplementation with 50 mg of zinc citrate and 3000 units of phytase increased the duration, efficacy and action of BoNT-A in the treatment of blepharospasm, hemifacial spasm and cosmetic facia rhytids [38]. Phytase enzymatically degrades phytates and other organic compounds that impair zinc absorption, thereby reducing their inhibitory effect [60]. Consequently, the co-administration of phytase with zinc supplements could enhance zinc bioavailability. However, independent scientific critique of the original Koshy et al. 2012 study raised methodological concerns (e.g., no baseline zinc status, ambiguous dosing, potential bias), and suggested a high level of clinical and scientific skepticism about the strength of its conclusions [38].

Recently, a new small randomized clinical trial in patients treated for excessive gingival display showed that zinc supplementation prior to BoNT-A injection could maintain the longer effect of BoNT-A and enhance its clinical efficacy [39].

A systematic review of the two abovementioned clinical studies confirmed some increase in botulinum toxin action or duration with zinc supplementation but emphasized that there are very few trials and that there is heterogeneity in their design (zinc forms, dosing, outcomes), which limits firm conclusions [40]. Overall, the existing evidence is insufficient to support a clinical recommendation for zinc supplements to enhance BoNT-A action on facial muscles. Therefore, new clinical studies are needed.

### 2.2. Magnesium

Magnesium functions as a fundamental regulator of neuromuscular transmission through multiple enzymatic and ion-channel pathways [61]. At the presynaptic level, magnesium is required for ATP synthesis and utilization, a process which is essential for acetylcholine vesicle mobilization and packaging. Furthermore, the key pharmacological property of magnesium is its antagonism of calcium channel activity; it competitively inhibits calcium influx through voltage-gated channels and thereby suppresses the triggering of vesicular exocytosis [62]. Consequently, elevated magnesium availability from dietary supplementation could theoretically create a complementary effect alongside BoNT’s SNARE cleavage mechanism, further dampening acetylcholine release at the neuromuscular junction [41]. This synergistic reduction in transmitter availability represents a biological mechanism by which magnesium supplementation might amplify or prolong BoNT’s clinical paralytic effect.

Nevertheless, this is still speculative without proper evidence. Hence, magnesium has a dual role as both an ATP cofactor, potentially supporting compensatory neurotransmitter synthesis, and a calcium antagonist, potentially enhancing BoNT paralytic action [42]. This creates competing pathways whose net effect in vivo cannot be predicted without proper studies.

### 2.3. Calcium

As aforementioned, the paralytic action of BoNT operates through degradation of SNARE complexes at the neuromuscular junction, which interrupts the release of acetylcholine. This mechanism is inherently reliant on calcium-mediated signaling because when calcium enters the presynaptic terminal through ligand-gated and voltage-sensitive calcium channels it initiates the exocytosis of acetylcholine vesicles into the synaptic cleft. Theoretically, if calcium abundance were augmented through dietary supplementation, or if calcium channel conductance were enhanced, the neuromuscular system might partially compensate for BoNT blockade of transmitter release by maintaining higher baseline acetylcholine availability. Nevertheless, experimental data from animal models of neurological dysfunction suggest an alternative picture [46]. When BoNT has been administered in conditions characterized by pathological elevation of intracellular calcium, the toxin has been observed to suppress both excessive calcium accumulation within myocytes and the expression of specific calcium channel subtypes. This finding indicates that BoNT’s therapeutic action may function independently or potentially downstream from whole-body calcium homeostatic balance, thereby diminishing the likelihood that supplemental calcium would substantially oppose its clinical effects.

However, dietary calcium supplementation could also possibly prolong BoNT’s therapeutic window through indirect physiological pathways. Calcium constitutes an essential cofactor for muscle contraction, relaxation and the anabolic remodeling of muscle tissue during periods of reduced activity, such as during BoNT-induced muscular attenuation [43]. Adequate calcium supply could foster the maintenance of myocellular architecture and energy production during the induced denervation, potentially stabilizing the microenvironment at the motor end-plate and slowing the rate at which BoNT is metabolized or cleared from the injection site [44]. Furthermore, calcium-dependent proteolytic enzymes govern intracellular protein degradation and modification pathways that may influence the persistence or elimination of BoNT and its biochemical sequelae [45]. Despite these plausible hypothetical scenarios, there are no available studies which systematically examined whether modifying dietary or serum calcium levels could alter the time to onset, magnitude, or longevity of BoNT clinical response in human subjects. To establish whether calcium supplementation constitutes a clinically actionable variable in BoNT treatment, rigorous intervention studies comparing standardized calcium supplementation regimens receiving uniform BoNT dosing protocols are needed.

### 2.4. Copper

Even though most focus regarding the BoNT paralytic effects is on the light chain’s dependency on zinc, recent biochemical investigations have revealed that the light chain has a susceptibility to group-11 and -12 transition metal cations, particularly copper [47,48]. Copper ions exhibit noncompetitive inhibition of the BoNT-A light chain effect with a dissociation constant, achieved through direct coordination with the critical cysteine residue on the enzyme surface. This metal-binding site operates outside the catalytic zinc-binding domain and the SNARE recognition sites, allowing copper to inhibit the enzyme without competing with the binding process. This mechanism was confirmed through X-ray crystallographic analysis and site-directed mutagenesis, demonstrating that cysteine mutation confers marked resistance to copper inhibition [47]. The noncompetitive nature of copper-mediated inhibition represents a distinct mechanistic advantage over traditional competitive inhibitors, as it circumvents the catalytic machinery rather than competing for binding sites.

Due to the abovementioned mechanism, copper supplementation delivered via the lipophilic ligand–copper complexes serves as a possibly effective strategy to overcome the inherent cellular impermeability of free copper ions and achieve intracellular delivery to cytosolic BoNT-A light chain. Copper complexes, particularly copper(II) bis(thiosemicarbazone) compounds such as Cu(GTSM) and copper(II) dithiocarbamate complexes such as Cu(MEDTC)_2_, penetrate neuronal membranes and undergo bioreductive activation, releasing free copper(I) through intracellular reduction mechanisms. Notably, in vivo efficacy was established in rodent models, where subcutaneous administration of copper complexes significantly extended survival time in mice challenged with BoNT-A [47]. Remarkably, the inhibitory activity of copper complexes appears independent of intracellular redox homeostasis. This suggests that endogenous copper may facilitate the intracellular trafficking of released copper to the cystein binding site on the BoNT-A light chain, thereby preserving SNARE complex integrity and neurotransmitter release capacity. These findings suggest that copper supplementation is an antagonist to BoNT-A action and could significantly diminish its effect. This hypothesis is further enhanced with the findings of another recent animal study which showed that copper supplementation reduced the efficiency of BoNT-A on rat masseter muscles [63].

### 2.5. Vitamin B

B vitamins, regarded as the B complex, consist of eight water-soluble essential compounds: thiamine (B1), riboflavin (B2), niacin (B3), pantothenic acid (B5), pyridoxine (B6), biotin (B7), folate (B9), and cobalamin (B12). These organic compounds play crucial roles in metabolism and require daily dietary replenishment due to urinary excretion [64]. Besides metabolic roles, they are also important in red blood cell formation and maintain nervous system function [49]. Dietary sources include plant-based foods and animal products, with vitamin B12 uniquely produced by bacteria in animal rumen, while others are synthesized in plants [65]. Deficiency in any of these can lead to significant health issues, particularly affecting the brain and nervous system. Worldwide estimates indicate high rates of inadequate intake, especially for riboflavin. Despite recommendations for a balanced diet, B vitamins are among the most commonly used dietary supplements, with over 33% of the population in the USA and many European countries using multivitamin preparations containing B vitamins [66]. Adverse effects and potential interactions with other medications are well recognized, especially when higher doses are used.

The potential interaction between vitamin B complex supplementation and the efficacy of botulinum toxin A administration was only investigated in an animal study on rat models. The researchers administered botulinum toxin A alone, vitamin B complex (B1, B6 and B12) before toxin injection, or both together and measured the compound muscle action potential (CMAP) as a marker of neuromuscular blockade. No significant difference in CMAP amplitude was observed between the groups, indicating that vitamin B supplementation does not diminish the neuromuscular blocking effect of botulinum toxin A. The findings suggest that concurrent use of vitamin B complex and botulinum toxin is safe and does not interfere with the therapeutic outcomes in experimental settings [51]. Contrary, an in vitro study tested whether can B3 be used as treatment against BoNT-A and the outcomes showed that BoNT-A effects were diminished when B2 was used in combination with a photosensitizer [50]. However, this was due to the photooxidation conducted in an in vitro study; as such, further studies are needed to address this issue.

### 2.6. Vitamin D

Vitamin D, a fat-soluble essential compound, is synthesized in the skin from 7-dehydrocholesterol upon exposure to ultraviolet B radiation and further metabolized in the liver and kidney to its active form, 1,25-dihydroxyvitamin D (1,25(OH)2D). Vitamin D is fundamental in the regulation of calcium and phosphorus homeostasis, bone health, immune function, and cellular growth, and is stored in adipose tissue, with excess excreted through bile and feces. Sunlight exposure is a major contributor to vitamin D status, while dietary sources include fatty fish, fish liver oils, egg yolks, fortified dairy products, and mushrooms. Deficiency in vitamin D can lead to significant health issues, particularly affecting bone health, immune function, muscle weakness and increasing the risk of non-communicable diseases such as cardiovascular and autoimmune disorders [67]. Worldwide estimates show that inadequate intake and deficiency of vitamin D are highly prevalent, particularly among individuals with limited sun exposure or insufficient dietary sources, prompting health strategies and supplementation for improving vitamin D status [68]. Supplementation with higher doses of vitamin D is generally well tolerated; however, adverse effects such as hypercalcemia may occur, particularly with prolonged use.

Vitamin D is crucial for skeletal muscle function, influencing muscle fiber contractility, strength, and growth through specific vitamin D receptors. Furthermore, it supports myocyte proliferation and regulates calcium influx into muscle cells, which is essential for contraction [54]. Recent research highlights the influence of vitamin D on muscle strength and its interaction with BoNT therapy. A study using surface electromyography (EMG) in patients with dynamic wrinkles found that those with sufficient vitamin D levels (above 30 ng/mL) had higher baseline muscle strength in the frontal muscle group compared to those with insufficient levels [55]. This increased muscle strength was associated with a higher required dosage of BoNT to achieve optimal muscle relaxation, suggesting that vitamin D status could possibly impact the clinical planning of BoNT treatments. Despite these differences, the durability of BoNT effects was similar between groups, indicating that vitamin D status primarily influences the initial dosage rather than the treatment duration. These findings underscore the potential importance of assessing vitamin D status in patients undergoing BoNT interventions, for both aesthetic or therapeutic applications targeting facial muscles [55]. However, it must be highlighted that further studies are needed to explore this association before making causal conclusions.

### 2.7. Vitamin E

Vitamin E (tocopherol), a fat-soluble compound with eight isoforms, has α-tocopherol as the predominant form in human plasma. It is synthesized by plants and absorbed in the small intestine via micelles; then, it is packaged into chylomicrons for transport to adipose tissue and liver, where it regulates membrane integrity, inhibits lipid peroxidation, and modulates immune and coagulation pathways. The main dietary sources include vegetable oils, nuts, seeds, and fortified foods, with excess stored in fat and eliminated through bile and urine [56]. Global data reveal widespread vitamin E intake insufficiency, with 82% of the population’s daily consumption below the adult recommended dietary allowance (RDA), especially where dietary staples like nuts and oils are scarce [69]. However, in developed countries, deficiency is rarely caused by a lack of nourishment, and the more common causes are pathologies like fat malabsorption syndromes, chronic cholestatic hepatobiliary disease, Crohn’s disease, and exocrine pancreatic insufficiency. Conversely, in developing countries, dietary insufficiency remains the primary cause [70]. Supplementation at doses below 1000 mg/day (the tolerable upper intake level) is well tolerated, as α-tocopherol overaccumulation is circumvented by urine and bile excretion. Higher doses, in contrast, pose a risk of coagulopathy, hemorrhagic stroke, and increased mortality, notably in patients on anticoagulants or with cardiovascular history [71].

There is no direct or indirect interaction between the vitamin E mechanisms and BoNT effects. However, several guidelines on BoNT recommend preoperative counseling to reduce bruising by discontinuing vitamin E alongside aspirin and NSAIDs for 10–14 days prior to treatment. This precaution targets vitamin E’s potential to impair platelet function and increase ecchymosis at injection sites, which id particularly relevant for aesthetic facial applications where cosmetic outcomes depend on minimizing hematomas [72]. No evidence suggests vitamin E affects botulinum toxin’s neuromuscular efficacy or duration [17].

### 2.8. Vitamin C

Vitamin C (L-ascorbic acid) is a water-soluble micronutrient with essential roles in the cellular antioxidant defense system. It is also critical for collagen biosynthesis, iron absorption, and immune system regulation. Because vitamin C cannot be synthesized endogenously in humans, continuous dietary intake is required to maintain adequate physiological levels. Major dietary sources of vitamin C include citrus fruits, berries, cruciferous vegetables, peppers, and tomatoes and it is primarily absorbed in the distal ileum of the small intestine through sodium-dependent active transport mechanisms [52]. Following absorption, it is preferentially distributed to metabolically active tissues, while quantities exceeding the renal threshold are excreted unchanged in urine [73]. Insufficient intake remains common, particularly in low-income populations and food-insecure regions, where deficiencies can result in impaired collagen synthesis, compromised immune function and delayed wound healing [74]. In healthy adults, daily intakes of 200–400 mg are typically sufficient to achieve plasma saturation, while intakes exceeding 400 mg/day provide minimal additional benefit due to saturable intestinal absorption and rapid renal clearance, which leads to urinary excretion of the surplus. While high-dose supplementation is generally considered safe, chronic excessive intake may induce gastrointestinal discomfort and increase urinary oxalate and uric acid levels [75].

Vitamin C functions through two mechanistically distinct pathways: as a cofactor for iron- and copper-dependent dioxygenases that enables collagen cross-linking, catecholamine synthesis and gene regulation, and through playing a role as an antioxidant that neutralizes reactive oxygen species through electron donation [52,53]. These mechanisms are biochemically independent from BoNT’s effects, as vitamin C’s roles in metalloenzyme cofactor activity do not intersect with botulinum toxin’s zinc protease function and vitamin C’s antioxidant properties do not modulate SNARE cleavage kinetics. Based on available data and the literature on vitamin C and BoNT, no direct biochemical interaction exists between these two substances. The two substances operate through entirely separate molecular pathways with no overlapping enzymatic cascades, allosteric regulation or metabolic interdependence, indicating an absence of any pharmacological interaction.

## 3. Conclusions

Even though there is a surge of popularity of BoNT for both aesthetical and medical purposes, it seems that the potential interaction with oral supplements is still under-researched. Besides zinc supplementation, due to zinc’s major role in BoNT’s mechanism of action, plausible and possible associations with other everyday oral supplements such as magnesium, calcium, vitamin B, vitamin D and vitamin E are all still insufficiently covered with research evidence. However, it is important to highlight that even zinc supplementation outcomes raised controversy. Consequently, additional investigations are needed to establish evidence-based protocols addressing this critical aspect which governs the dynamics of BoNT efficacy.

## Figures and Tables

**Table 1:** Summary showing the level of evidence regarding BoNT and dietary supplements interaction.

Supplement	Foundational Evidence	Level-5 Evidence	Level-4 Evidence	Level-3 Evidence	Level-2 Evidence	Level-1 Evidence
Zinc	Zinc dependent SNARE cleavage essential for BoNT-A activation [37]	/	/	/	RCT reports of enhanced efficacy in facial applications [38] Supplementation prior to BoNT-A injection maintained longer effect [39]	Confirmed some increase in BoNT-A action/duration but heterogeneity in design limits firm conclusions [40]
Magnesium	Theoretical synergy: dual role as ATP cofactor and calcium-channel antagonist to enhance BoNT paralytic effect [41,42]	/	/	/	/	/
Calcium	Theoretical mechanisms: potential compensatory role via exocytosis or delayed BoNT clearance [43,44,45]	/	Animal data show BoNT suppresses elevated intracellular calcium, suggesting BoNT acts independently [46]	/	/	/
Copper	Noncompetitive inhibition of BoNT-A light chain through cysteine-binding site independent of zinc domain [47,48]	Antagonizes BoNT-A in rodent survival models [47]; supplementation reduced BoNT-A efficiency [48]	/	/	/	/
Vitamin B	B vitamins essential for neurotransmitter synthesis but biochemically independent of BoNT SNARE cleavage [49]	In vitro B3 study showed antagonism but requires photooxidation [50]	Animal study showed B1, B6, B12 have no effect on BoNT-A blockade [51]	/	/	/
Vitamin C	Copper and irondependent dioxygenase cofactor activity and antioxidant functions are biochemically independent of BoNT zinc protease mechanism [52,53]	/	/	/	/	/
Vitamin D	Influences baseline muscle strength and neuromuscular status but independent of SNARE cleavage [54]	/	/	Associated with higher baseline frontal muscle strength, requiring higher BoNT doses [55]	/	/
Vitamin E	No direct or indirect interaction with BoNT protease activity or SNARE cleavage [56]	/	/	/	/	/

## Data Availability

No new data were created or analyzed in this study. Data sharing is not applicable to this article.
